# Differential A1/A2 β-casein (*CSN2*) gene-derived allelic and genotypic frequencies across Ecuadorian exotic dairy cattle breeds

**DOI:** 10.3389/fvets.2025.1616426

**Published:** 2025-07-09

**Authors:** Luis F. Cartuche-Macas, Jorge F. Navarrete-Mera, Miguel A. Gutiérrez-Reinoso, Manuel García-Herreros

**Affiliations:** ^1^Escuela Superior Politécnica Agropecuaria de Manabí Manuel Félix López (ESPAM), Carrera de Medicina Veterinaria, Calceta, Ecuador; ^2^Asociación Holstein Friesian del Ecuador (AHFE), Quito, Ecuador; ^3^Laboratorio de Biotecnología Animal, Departamento de Ciencia Animal, Facultad de Ciencias Veterinarias, Universidad de Concepción (UdeC), Chillán, Chile; ^4^Facultad de Ciencias Agropecuarias y Recursos Naturales, Carrera de Medicina Veterinaria, Universidad Técnica de Cotopaxi (UTC), Latacunga, Ecuador; ^5^Instituto Nacional de Investigação Agrária e Veterinária (INIAV), Santarém, Portugal; ^6^CIISA-AL4AnimalS, Faculty of Veterinary Medicine, University of Lisbon, Lisbon, Portugal

**Keywords:** *CSN2* gene, beta-casein, A1/A2, genetic diversity, geographic location, dairy cattle

## Abstract

Recently, a growing interest in dairy cattle selection has been triggered due to specific genetic variations of the β-casein (*CSN2*) gene which was associated to several human disorders. The aim of the present study was to evaluate the A1/A2 β-casein (*CSN2*) gene-derived allelic and genotypic frequencies in five Ecuadorian dairy cattle breeds to determine the genetic diversity of the A1/A2 β-casein locus. Genomic Deoxyribonucleic Acid (gDNA) was extracted from hair samples collected from different dairy cattle breeds, namely Holstein Friesian (HF), Brown Swiss (BS), Jersey (J), Gyr (G), and crossbreds (CB) raised commonly in seven different regions in order to identify A1 and A2 variants. Genomic determination of the β-casein (*CSN2*) gene variants was carried out evaluating the Exon 7 region in order to identify and confirm the accuracy of the A1/A2 β-casein (*CSN2*) gene-derived polymorphisms related to the genotypes and the allelic profiling. A2 allele frequencies were determined as 0.628, 0.643, 0.727, and 0.644 in HF, BS, J, and CB, respectively. In contrast, the lowest A1 allele frequency (0.145) as well as the greatest A2 allele frequency (0.855) was detected in G. No significant differences for allelic frequency were observed among breeds (*p* > 0.05). Homozygous A1 genotype frequency in HF, BS, J, and CB was 0.140, 0.110, 0.050 and 0.150, respectively. Interestingly, the A1A1 genotype was absent in G (*p* < 0.05). A1A2 genotypes were 0.290, 0.490, 0.450, 0.290, and 0.420 in HF, BS, J, G, and CB, respectively. Finally, the A2A2 genotype was 0.400, 0.400, 0.500, and 0.460 in HF, BS, J, and CB, respectively. However, the greatest A2A2 genotype frequency was observed in G (0.710). No deviation from the Hardy–Weinberg equilibrium was found in any breed in terms of β-casein gene (*p* > 0.05). Moreover, the results revealed a geographically related distribution of A1/A2 allele and genotype frequencies of β-casein (*CSN2*) gene. In terms of genetic diversity a differential distribution of heterozygosity was observed among breeds being average Ho, He, uHe, and *F* values determined as 0.429, 0.438, 0.439, and 0.022, respectively. In conclusion, the β-casein (*CSN2*) gene was polymorphic in all the cattle breeds analyzed. The A1/A2 allele and genotype frequencies varied depending on the breed and the geographic region which may be conditioned by the specificities related to different breeding selection programmes. A2 allele and A2A2 genotype frequency were particularly greater in Gyr cattle which have a great potential for A2 milk production without compromising genetic diversity.

## 1 Introduction

The introduction of new exotic dairy cattle breeds in Ecuador during the second half of the 20th century opened up new opportunities for the rural population and the national dairy markets (1, 2). During the last decade, the genetic selection of dairy cattle has been crucial for increasing milk quality and productivity; however, undesirable effects such as gastrointestinal disorders resulting from the consumption of raw or processed milk have been observed in the Ecuadorian population (3–5). Several studies reported more than twenty genes associated with essential milk trait composition (6–8). The sequence analysis of the milk protein *CSN2* gene (exon 7 regions in chromosome 6) revealed different genotypes and allele frequencies (e.g., A1, A2, A3, B, among others) of the β-casein variants which were intensively genotyped in dairy cattle for selection purposes (5, 9). The β-casein protein is composed of 209 aa being the 2nd most abundant protein in milk (10, 11). During the last years, the determination of genetic variations (polymorphisms) of bovine β-casein protein has become a common practice in dairy cattle being the A1 and A2 alleles the most common among the 13 alleles identified (12). One of the main reasons was related to the A1 allele variant which has been considered as risk factor for several heart diseases, diabetes mellitus (type 1), autoimmune diseases, and milk intolerance issues in humans (13). These issues are derived from the release of the bio-active opioid β-casomorphin-7 (BCM-7) during the histidine proteolytic digestion of A1 monomers (14). Several studies reported that the A1 allele variant appeared as a mutation of the A2 allele resulting in a polymorphism mutation at the position 67 of the *CSN2* gene in *Bos taurus* cattle (15–17). Basically, there is a change of adenine for a cytosine in the amino acid chain, and as a consequence, the mutation causes the exchange of proline for histidine (5). The export of specialized genetics (semen, embryos and live animals) of different dairy cattle breeds, caused the A1 allele variant to widespread in most parts of the world (3, 18). On the contrary, the A2 allele variant has been considered as non-problematic regarding health-related issues (19). Thus, the A2 milk can be obtained from cows carrying the A2A2 genotype of the *CSN2* gene, being therefore the cows owning the genotypes A1A1 and A1A2 undesirable for row milk consumption. However, although there are studies suggesting that milk from cows with the A2 genotype is easily digestible, it should be noted that, in cheese production, milk containing A2 alleles is not recommended, as it is associated with a deterioration in the technological characteristics of the milk and, consequently, with a less efficient cheese-making process (20, 21). Moreover, the allele and genotype frequencies vary according to the cattle breed (5, 17). Thus, there is an imperative need of developing genetic selection programs for breeding dairy cattle intended for the consumption of raw milk with the aim of increasing the A2 allele variant frequency irrespective of the country considered (22). In Ecuador, according to INEN-ESPAC (23) the distribution of dairy cattle breeds showed the Holstein, Jersey and Brown Swiss as the main dairy breeds in the highlands and the Jersey, Brown Swiss, and Gyr breeds in the coast and the Amazon region (23). On the other hand, the Ecuadorian Ministry of Agriculture and Livestock (24) has established through ministerial agreement No. 061–2021 the regulations for the certification of A2A2 genotype, which aims to promote A2A2 milk production through the identification of individuals and herds at national level (24).

In view of the above, the genetic characterization programs in dairy cattle are crucial not only for increasing the milk quality and yield but also for assuring the optimal milk composition and characteristics which are related to human health (25, 26). Thus, the molecular identification and analysis of milk-related genes as *CSN2* including different polymorphisms, contribute to increase the efficiency of cattle selection programmes (27, 28). There are currently no studies in the Ecuadorian dairy cattle population and its relationship to different genotypes and allele frequencies of the milk protein *CSN2* gene (29, 30). Therefore, the aim of the present research was to characterize the *CSN2* gene to determine the allelic (A1 and A2) and genotypic (A1A1, A1A2, and A2A2) frequencies of exotic dairy cattle breeds and crossbreds in Ecuador. This will provide a baseline on the knowledge of the *CSN2* gene that will be undoubtedly useful for the different dairy cattle producers and associations with the aim of improving the cattle breeding and selection programmes.

## 2 Materials and methods

### 2.1 Ethical statement

The study was conducted according to the guidelines of the Declaration of Helsinki and following the Code of Ethics for animal experiments as reflected in the ARRIVE guidelines available at http://www.nc3rs.org.uk/ARRIVEchecklist (Accessed on 10 August 2024). However, the present research did not require any ethical review and approval by a specific Bioethics Committee for the use of experimental animals, since the hair sample collection is considered a non-invasive method and were obtained during routine diagnostic and health control services in each farm. Therefore, none of the animals were handled or restricted at any time for this study. Then, the studies were conducted in accordance with the local legislation and institutional requirements.

### 2.2 Animals, sample collection and geographical location

Hair root samples (at least 20–25 hair roots per animal) were collected for DNA extraction from the hair follicles (bulbs found at the base of the hair follicle). The samples (*n* = 1,599) were collected individually from ears avoiding contamination and assuring sufficient quantity and quality of DNA. The collection was carried out by the technical staff (veterinarian) of each farm for routine diagnostic and health control services. The samples belonged to farms located in seven provinces from two geographical regions [West region: Santo Domingo, Manabí, and Guayas (Coast) and East region: Pichincha, Cotopaxi, Tungurahua, and Azuay (Highlands)] in Ecuador. Four pure-bred [Holstein Friesian (*n*: 701), Gyr (*n*: 258), Jersey (*n:* 86), and Brown Swiss (*n*: 230)] and a cross-bred [always Holstein Friesian x another dairy breed (*n*: 324)] populations were analyzed for the detection of A1/A2 β-casein-derived allelic and genotypic frequencies using molecular techniques.

### 2.3 β-casein characterization using genomic analysis

The characterization of β-casein (*CSN2*) gene was carried out by genomic analysis. Thus, the individuals were studied with genomic tests using the Illumina Bovine SNP50 BeadChip (Clarifide–Zoetis, San Diego, CA, USA and Igenity-Neogen, Lansing, MI, USA). For the genotyping sampling, the Gene Max kit (TSU, ALLFLEX, Kalamazoo, MI, USA) was used to take samples from each cow ear (pinna). A chip with 12,000 DNA markers (12 K) was used, which provides the most relevant information regarding production and milk-type-related characteristics with a ~ 70% reliability based on a panel of 50,000 (HD50K) genetic markers from the Animal Improvement Programs Laboratory (AIPL) (Zoetis–Clarifide, San Diego, CA, USA) and the Animal Laboratory (Neogen– Igenity, Lansing, MI, USA). The information predicted mutations of the CSN2 gene in bovine and have been widely used for the characterization of elite sires and dams in the dairy industry (Clarifide™ Plus, Zoetis Genetics, San Diego, CA, USA and Igenity® Plus-Neogen, Lansing, MI, USA). The initial genomic analysis (screening) allowed identifying and characterizing those individuals related to different variants for the *β*-casein (*CSN2*) gene (A1A1, A1A2 and A2A2) to later determine the genotype and allele frequencies. Other β-casein (*CSN2*) gene variants such as A3, A4, B, C, D, E, F, G, H1, H2, I, J, and K were not considered for the present study. The distinction between A1 and A2 genotype was carried out, as the β-casein variants were categorized into two groups based on the amino acid at position 67 of the protein: Type A1 (histidine) and Type A2 (proline).

### 2.4 Frequency-based population genetic parameters related to A1/A2 β-casein (CSN2) gene

Frequency-based population genetic parameters related to A1/A2 β-casein (*CSN2*) gene of different dairy cattle breeds raised in Ecuador are showed in Supplementary Table S1. Each population genetic indicator was based on methodologies carried out previously by different authors. Then, the following parameters were evaluated: (i) allelic frequency (31); (ii) genotypic frequency (Haploid Data) (32); (iii) Hardy–Weinberg disequilibrium (32); number of alleles (33); number of effective alleles (34); Shannon diversity index (34); observed heterozygosity (32); expected heterozygosity (31); unbiased expected heterozygosity (35); fixation index (31); Nei unbiased genetic distance (32); and finally, paired Fst (36). The corresponding variables, formulas, value ranges, parameter descriptions and the reference sources are displayed in Supplementary Table S1.

### 2.5 Statistical analyses

Statistical differences among breeds and regions were analyzed using the excel plugin GenAlEx 6.5 (35). To understand the distribution of *CSN2* gene across populations, the allele and genotype frequencies were calculated. Based on the obtained results, the population differentiation and selection effects Hardy–Weinberg disequilibrium, number of alleles, number of effective alleles, Shannon diversity index, observed/expected and unbiased expected heterozygosity and fixation index were obtained. Finally, the comparison between populations was tested by paired unbiased Nei genetic distance and paired Fst. Significance level was set at *p* ≤ 0.05.

## 3 Results

### 3.1 A1/A2 β-casein-derived allelic and genotypic frequencies in different dairy cattle breeds

Differential pattern of the A1A1, A1A2, and A2A2 genotype frequencies was observed across Brown Swiss, Cross-bred, Gyr, Holstein-Friesian, and Jersey cattle breeds (Table 1). The greatest frequency was observed for A2A2 genotype in Gyr (0.710) and Jersey (0.500), followed by heterozygous A1A2 in Brown Swiss (0.490) and Holstein-Friesian (0.460) and homozygous A1A1 genotype in Cross-bred (0.150) and in Holstein-Friesian (0.140). Interestingly, the A1A1 genotype was absent in G (*p* = 0.006) (Table 1). Regarding frequency of A1 allele, the highest value was observed in Holstein Friesian (0.372) followed by Brown Swiss (0.357) and Cross-Bred (0.356). Finally, with regard to the frequency of A2 allele, the highest value was observed in Gyr (0.855), followed by Jersey (0.727). The lowest values of A2 allele were observed in Holstein Friesian (0.628), in Brown Swiss (0.643) and in crossbred (0.644), respectively. No significant differences for allelic frequency were observed among breeds (*p* > 0.05) (Table 2).

**Table 1:** A1/A2 β-casein-derived genotypic frequencies in different dairy cattle breeds raised in Ecuador.

Breed	Genotype frequency	A1A1 (n)	A1A2 (n)	A2A2 (n)	*p*
Brown Swiss	Observed	0.110 (26)	0.490 (112)	0.400 (92)	0.353
	Expected	0.130 (29.22)	0.460 (105.54)	0.410 (95.22)	
Cross-Bred	Observed	0.150 (48)	0.420 (135)	0.460 (141)	0.098
	Expected	0.130 (41.17)	0.460 (148.65)	0.410 (134.17)	
Gyr	Observed	0.000 (0)	0.290 (75)	0.710 (183)	0.006
	Expected	0.020 (5.45)	0.250 (64.09)	0.730 (188.45)	
Holstein Friesian	Observed	0.140 (98)	0.460 (325)	0.400 (278)	0.847
	Expected	0.140 (96.80)	0.470 (327.39)	0.390 (276.81)	
Jersey	Observed	0.050 (4)	0.450 (39)	0.500 (43)	0.189
	Expected	0.070 (6.42)	0.400 (34.16)	0.530 (45.42)	
All Breeds	Observed	0.110 (176)	0.430 (686)	0.460 (737)	0.389
	Expected	0.110 (168.46)	0.440 (701.09)	0.460 (729.46)	
Highlands	Observed	0.130 (161)	0.450 (543)	0.410 (495)	0.532
	Expected	0.130 (156.01)	0.460 (552.98)	0.410 (490.01)	
Coast	Observed	0.040 (15)	0.360 (143)	0.610 (242)	0.274
	Expected	0.050 (18.70)	0.340 (135.58)	0.610 (245.70)	

n, number of animals.

**Table 2:** A1/A2 β-casein-derived allelic frequencies in different dairy cattle breeds raised in Ecuador.

Breed	Allele frequencies	
	A1	A2
Brown Swiss	0.357	0.643
Cross-Bred	0.356	0.644
Gyr	0.145	0.855
Holstein Friesian	0.372	0.628
Jersey	0.273	0.727
All breeds	0.325	0.675
Highlands	0.361	0.639
Coast	0.216	0.784

### 3.2 A1/A2 β-casein-derived allele and genotype frequencies in dairy cattle breeds and geographical location

Allelic and genotypic frequency profiles were also generated from samples in different dairy cattle breeds raised in different geographical regions (Highlands and Coast; Tables 1, 2). Specific pattern of allelic and genotype frequency distribution was observed across groups based on geographical location. For the samples screened, the frequency of A2 allele and A2A2 genotype was the highest in the Coast region, being 0.784 and 0.610, respectively. On the contrary, the frequency of A1 allele and A1A1 genotype was the highest in the highlands region, being 0.361 and 0.130, respectively, compared to the Coast region where the frequencies for A1A1 genotype and A1 allele were 0.040 and 0.216, respectively (Tables 1, 2). Finally, the A1A2 genotype was predominant in the highlands region (0.450) compared to the coast region where A1A2 genotype frequency was lower (0.360). No significant differences for allelic frequency were observed between geographical regions (*p* > 0.05).

### 3.3 Genetic diversity indices in dairy cattle breeds related to A1/A2 β-casein (CSN2) gene

Genetic diversity indices of different dairy cattle breeds raised in Ecuador are showed in Table 3. The allele and genotype-derived results obtained in the current study revealed that a significant level of genetic diversity was observed in dairy cattle breeds. All the selected loci were found 100% polymorphic in all dairy cattle breeds except in Gyr, where no A1 allele and A1A1 genotype was found. The number of alleles observed at each locus serves as an indicator of genetic diversity, which directly influences the differentiation of dairy cattle breeds as each locus exhibited different number of alleles; however, in the present study the number of observed alleles (Na) detected all dairy cattle breeds was just two (A1 and A2) (Table 3). The average number of effective alleles (Ne) varied from 1.331 in Gyr breed to 1.877 in Holstein Friesian breed with mean average Ne across cattle breeds of 1.781. Among all the breeds studied, highest detected heterozygosity (Ho) was found in Brown Swiss breed (0.487) while lowest was observed in Gyr breed (0.291). Further, average observed heterozygosity across the whole breeds was found to be 0.429 (Table 3). The expected heterozygosity (He) was found either approximately similar than Ho in all the breeds studied, except for Jersey breed where was much less (0.397) compared to Ho (0.453). Finally, the highest value related to unbiased expected heterozygosity (uHe) was observed in Holstein-Friesian breed (0.467) while the lowest was detected again in Gyr breed (0.249). The fixation index (F) to detect differences in the dairy breed related to genetic structure indicated that the more fixed positive value was detected in crossbred individuals (0.092) while the less and negative *F* value was observed in Gyr breed (−0.170). The negative F value in Brown Swiss, Gyr and Jersey breeds indicated the absence of inbreeding in the analyzed breeds.

**Table 3:** Genetic diversity indices in different dairy cattle breeds and geographical regions related to A1/A2 variants of β-casein (CSN2) gene.

Breed	N	Na	Ne	I	Ho	He	uHe	F
Brown Swiss	230	2.000	1.848	0.651	0.487	0.459	0.460	−0.061
Cross-Bred	324	2.000	1.848	0.651	0.417	0.459	0.460	0.092
Gyr	258	2.000	1.331	0.415	0.291	0.248	0.249	−0.170
Holstein Friesian	701	2.000	1.877	0.660	0.464	0.467	0.467	0.007
Jersey	86	2.000	1.659	0.586	0.453	0.397	0.397	−0.142
All breeds	1,599	2.000	1.781	0.630	0.429	0.438	0.439	0.022
Highlands	1,199	2.000	1.856	0.654	0.453	0.461	0.461	0.018
Coast	400	2.000	1.513	0.522	0.358	0.339	0.339	−0.055

N, Sample Size; Na, No. Alleles; Ne, No. Effective Alleles; I, Information Index; Ho, Observed Heterozygosity; He, Heterozygosity expected; uHe, Unbiased Expected Heterozygosity; F, Fixation Index.

### 3.4 Genetic diversity indices related to β-casein A1/A2 variants and geographical location

Genetic diversity indices of different geographical locations where different dairy cattle breeds are raised in Ecuador are showed in Table 3. In the present study the number of observed alleles (Na) detected was just two (A1 and A2). The average number of effective alleles (Ne) varied from 1,513 in the coast to 1,856 in the highlands region. The highest observed heterozygosity (Ho) was found in the highlands region (0.453) while lowest was detected in the coast (0.358). In the other hand, he expected heterozygosity (He) was pretty similar in the highlands compared to Ho; however, the coast region was lower (0.339) compared to Ho (0.358). Finally, the highest value related to unbiased expected heterozygosity (uHe) was observed in the highlands population (0.461) while the lowest was detected again in the coast population (0.339). The fixation index (F) indicated a positive value (0.018) in the highlands cattle population, while a negative F value was observed in the coast population (−0.055).

### 3.5 Differentiation and genetic relationship among dairy cattle breeds (Fst: fixation index and DA: unbiased Nei genetic distance)

The pair-wise Fst values of different dairy cattle breeds are displayed in Table 4. The Fst values, which indicate breed differentiation, demonstrated that 4.74% of the total genetic variability resulted from allelic differences among the different breeds. The remaining 95.26% of genetic variability corresponded to differences among individuals within the breed. The Fst values ranged from 0.000 to 0.067. This indicates the least differentiation among Holstein Friesian, Brown Swiss and Crossbred individuals, while the greatest divergence was observed between Holstein Friesian-Brown Swiss-Crossbred vs. Gyr breed. The pair-wise DA values for stabilizing the genetic relationship among the different dairy cattle breeds are showed in Table 4. The lowest DA value (shortest distance) was observed among the Holstein Friesian-Brown Swiss-crossbred (0.000) followed by Jersey-Brown Swiss (0.007) and Jersey-crossbred (0.008). The Gyr was the most distant breed, displaying the largest DA when compared to the Holstein Friesian (0.067), to the cross-bred (0.056) and to the Brown Swiss (0.055) (Table 4).

**Table 4:** Pairwise unbiased Nei distance (below diagonal) and Fst (above diagonal) among different dairy cattle breeds.

Breed	Brown Swiss	Cross-Bred	Gyr	Holstein Friesian	Jersey
Brown Swiss	–	0.000	0.059	0.000	0.008
Cross-Bred	0.000	–	0.059	0.000	0.008
Gyr	0.055	0.056	–	0.067	0.025
Holstein Friesian	0.000	0.000	0.067	–	0.011
Jersey	0.007	0.008	0.015	0.013	–

## 4 Discussion

The present research aimed to study the distribution of the beta-casein gene (*CSN2*) genotypes and alleles in the main dairy cattle breeds in Ecuador. The first studies related to the *CSN2* gene were carried out more than two decades ago; however, the market demand for milk production with specific characteristics related to A1/A2 variants began only a decade ago, mainly in countries such as New Zealand, Australia, Canada, United States, among others (37, 38). Due to the advances made in the cattle selection using molecular techniques (genotyping), there is currently great interest in different countries in selecting sires and cows for genetic improvement related to the fixation of the A2 allele in both sexes (12). In this way, AI and genetic companies for cattle breeding and selection started to select sires as carriers of the beta-casein *CSN2* gene in favor of the A2 allele promoting the use of semen from sires carrying this allele (39–41).

It has been previously described that the A2 allele mutation to A1 has occurred in the Holstein Friesian breed (5). The allele A1 frequency in Holstein Friesian has been described as high by different studies (9, 40). However, the intense selection in this breed could influence the higher A1 allele frequency observed which could be related to individuals producing higher milk yields (40). In the present study, the A2 allele frequency was slightly lower in Holstein Friesian and Brown Swiss cattle compared to the other breeds, with the exception of the Jersey and Gyr breed where the A2 allele frequency was higher (42, 43). In the case of Holstein Friesian, the A2 allele frequency was similar to that observed in other countries such as Croatia, Italy and Sweden (40), but lower than in Chile, Slovakia, and Greece (38) (Supplementary Table S2). In this study the A2 allele frequency observed in Holstein Friesian was higher than that observed in Peru, Spain, Turkey, Italy (north), and China (22). Finally, the A2 allele frequency in this breed was 1.5 to 2 times higher in Ecuador than in India, Russia, and Pakistan (40). In addition, due to the great expansion of this breed worldwide, the *CSN2* gene mutation has been spread as well, partly due to the use of semen from countries that maintain solid genetic improvement programs and the massive export of genetic material from live sires and embryos (44). The high A2 allele frequency in Ecuador could be due to the fact that genetic material has been imported mainly from A2A2 genotyped sires. For example, in Ecuador out of 311 imported sires born between 2016 and 2021, a total of 151 owned the A2A2 genotype and 130 sires the A1A2 genotype (45). This fact indicates that in recent years the application of new technologies in favor of the selection of individuals carrying the A2A2 genotype is steadily increasing. In addition to the genetic influence of European Holstein Friesian sires for the selection programs related to the A2A2 genotype in Ecuador, the genetic material also included several genes for improving the milk protein profile (45).

The prevalence of the A2 allele in the Guernsey and Jersey breeds was more frequent than in other European breeds (46, 47). In the present study, the A2 allele frequency was higher for all breeds analyzed, except for the Gyr (48). The A2 allele frequency obtained was similar to those observed in countries such as Chile, Mexico, Turkey, Japan and Sweden in the same breed (17) and higher than those observed in Russia and India, even tripling the value observed in the Pakistani Jersey (49) (Supplementary Table S2). This fact could be due to the fact that in developed countries, the use of ART’s using genetic material from Jersey sires of US, Canadian or European origin has been much more accentuated and, therefore, they benefit indirectly from the increased population of individuals carrying the A2 allele (50). Thus, the low use of imported semen together with the lack of local genotyping services for the identification of A2A2 genotype carriers led to the fact that there was no genetic improvement in favor of this genotype in less developed countries. Therefore, this fact could possibly favor that the A1 allele frequency has been maintained at above average levels in non-developed countries. In any case, according to the results obtained in the present study, the Ecuadorian Jersey breed seems to have a great potential to increase the A2 allele frequency, both within the breed itself, as well as within crossbred individuals using the Jersey breed. However, Ecuador, similarly to currently other less developed countries, lacks strict selection and genetic evaluation programmes for controlling and selecting individuals based on the *CSN2* gene and A1/A2 variants. In the case of the Brown Swiss breed, it has been observed that the A2 allele frequency reached the value of 0.785 and the frequency of the A2A2 genotype reached the value of 0.62 (51, 52). These frequencies observed in the Brown Swiss population were greater than those obtained for the same breed in Ecuador. The difference could be due to the fact that there has not been a specific selection program to increase the A2 allele frequency in Ecuador, which also explains the high A1 allele frequencies observed in the Ecuadorian Brown Swiss cattle population. In recent years, due to the genetic influence of European Brown Swiss sires (3, 53), the allele frequency in favor of A2 could probably increase in the coming years. Regarding the A2 allele frequencies published in the scientific literature, this allele has been variable within the European cattle breeds (*Bos taurus*) (54). However, the A2 allele frequency in the Indian zebu breeds (*Bos indicus*) was predominant (55) (Supplementary Table S2). Regarding Gyr breed, in the present study the A2 allele frequency was lower than that observed in Brazil (country of origin of this breed) (48). Taking into account that the genetic resources entering Ecuador came from Brazil, the fact that in the present study the frequency values were lower could be due to the Gyr breed entered Ecuador for the first time in the 1960s (30). From that moment on, the reproduction was carried out without control using a reduced number of individuals. In addition, most of the breeders used Gyr for crossbreeding with Holstein Friesian individuals producing the Girolando (Gyr x Holstein Friesian) synthetic breed, which in many cases remained within the herd jointly (56). So, it was possible that these Girolando individuals introduced the Holstein Friesian A1 allele into the Gyr breed in a higher proportion in Ecuador. Thus, just since 2023 the Ecuadorian Gyr-Girolando Association registered the first foundational population of this breed, which allowed better control of both the A2 allele and the A2A2 genotype through controlled genetic selection programs. The results obtained in the present study indicate that the Ecuadorian Gyr breed has prospects of increasing the frequency of the A2 allele in a few generations by selecting genotyped sires and dams as happened in Australia between 2000–2023, which managed to increase the A2 allele frequency from 0.32 to 0.52 (57).

With regard to the genetic diversity, it should be taken into account that the expected heterozygosity (He) quantifies the equality of allele frequencies in the loci (58). The genetic population structure can be evaluated when He was compared to the observed heterozygosity (Ho) (59). Thus, when analyzing the Hardy–Weinberg equilibrium and the Ho value is greater than the He, the population presents an excess of heterozygotes compared to what’s expected. On the contrary, when Ho is lower than He value, the population present a deficit of heterozygotes. Thus, in the present study an excess was observed in the dairy breeds evaluated, specifically in Brown Swiss, Gyr and Jersey, while a deficit was observed in crossbred and Holstein Friesian breed. The highest heterozygosity was observed in the Brown Swiss, and the lowest in the Gyr breed. He value in Holstein Friesian was the highest and the lowest was observed in the Gyr breed. In the case of Ecuadorian Holstein Friesian, the He value was higher than that observed in the population of the same breed in Ukraine (He = 0.343) (60) and similar to the local Black-White breed in the same country (He = 0.45–0.49) (9). Moreover, the He value was similar when compared to the Holstein Friesian from Mexico (Ho = 0.464) (61) from Chile (He = 0.375) (62) and from Turkey (He = 0.499) (17). Both He and Ho values found in the Ecuadorian Jersey breed (Ho = 0.453; He = 0.397) were greater than those observed in the Turkish Jersey cattle population (Ho = 0.262; He = 0.262) (50) in which the two variables (He and Ho) were in Hardy–Weinberg equilibrium. Thus, the values obtained in the same breed in Ecuador were similar to the Chilean Jersey breed population (He = 0.394) (62) and similar to those observed in Turkish Jersey (He = 0.338) (17). Regarding the Gyr breed, the He value (0.248) was greater than that described by Khan et al. (40), which was 0.06 (the presence of A1A1 was null) while the A1A2 genotype was 0.07. Regarding the Ecuadorian Brown Swiss breed, the He value (0.459) was similar to that obtained in the Turkish Brown Swiss (He = 0.466) (17).

The highest F (Fixation Index) value observed in the present study was 0.092 in crossbred and 0.007 in the Holstein Friesian breed, which could indicate the absence of active formation processes (e.g., selection, population size, and inbreeding, among others) and therefore, corresponds completely to the Hardy–Weinberg equilibrium of these populations (63). The F (Fixation Index) values obtained in the present study from the Brown Swiss, Jersey and Gyr breed were negative, although quite close to 0 (could even be considered = 0), therefore, there were also in Hardy–Weinberg equilibrium (9). Moreover, this could also indicate an excess of heterozygotes in the evaluated populations as established in the Polish White-Backed, Polish Red, Polish Holstein-Friesian, and Simmental populations (60). The Ne values found in the present study (1.331 to 1.848) would indicate an average level of polymorphism of the locus relative to the classical biallelic system (31). In general, the Fst and Pairwise Unbiased Nei Distance values observed in the present study were relatively low which would indicate a low differentiation and genetic distance among the evaluated breeds similar to those observed in other studies (60).

When analyzing the allelic frequencies by geographic location, a high frequency of the A1 allele was observed in the highlands region. In the same way, a high frequency of heterozygous individuals (A1A2) was observed maybe due to the high presence of the Holstein Friesian breed in this area which is characterized by having this allelic and genotypic profile. However, in the coast region, the predominant allelic and genotypic frequency was A2A2. This may be due to the fact that in that area there was a predominance of Gyr, Jersey, and crossbred individuals, which were characterized, especially the first two, by a high A2A2 allele frequency. Therefore, this difference in allelic and genotypic frequencies between regions could also be due to the unequal distribution of dairy cattle breeds. Thus, in the highlands the presence of Holstein Friesian was higher (23) while on the coast region the presence of Jersey, Gyr, Girolando and crossbreds with Brahman was predominant. As a result, it could be considered that the human population in the coast region has been consuming greater quantities of milk from individuals owning the A2A2 genotype which would be very beneficial to avoid digestibility problems derived from the consumption of milk from individuals with higher A1 allele frequencies that is predominant in the highlands region. Finally, regarding the genetic diversity parameters by region, no differences were observed in terms of genetic distances, so it could be considered that the global population of individuals maintained a balance among them.

## 5 Conclusion

This study suggests that the allelic and genotypic frequencies of A1/A2 variants of β-casein (*CSN2*) gene were variable in exotic reared dairy cattle breeds in Ecuador. A relatively high frequency of β-casein A2 allele was observed Gyr and Jersey cattle for A2 milk production since they carry a favorable A2 genotype compared to the other breeds. The polymorphisms of β-casein (*CSN2*) gene revealed a geographically associated distribution of allelic and genotypic frequencies. The differential frequencies observed between the West (Coast) and the East (Highlands) suggests that the genetic variation may be a result of the introduction of dairy cattle adapted to different climatic conditions as well as geographic artificial selection. Interestingly, an introgression of taurine genes into indicine breeds could have contributed to the increase of frequency of β-casein A1A2 genotype in Gyr which was higher than that obtained in other countries. Finally, the information on A1/A2 variants of β-casein (*CSN2*) gene could be used in genetic selection programmes for different dairy cattle breeds raised in Ecuador increasing A2 allele which was associated with the production of healthier milk for human consumption.

## Data Availability

The datasets presented in this study can be found in online repositories. The data set that supports the findings of the present research is available online in Zenodo repository (10.5281/zenodo.15114168).
