# Supplementary material for: Differential A1/A2 β-casein (CSN2) gene-derived allelic and genotypic frequencies across Ecuadorian exotic dairy cattle breeds
Source: Front Vet Sci. 2025 Jul 9;12:1616426. doi: 10.3389/fvets.2025.1616426 (PMC12285589; doi:10.3389/fvets.2025.1616426)
Supplement: Supplementary file 1 [file Table_1.docx]

Supplementary Material

# Supplementary Tables

**Table S1.** **Definition of frequency-based population genetic parameters in dairy cattle breeds related to A1/A2 β-casein (*CSN2*) gene**

| **Parameter** | **Variable** | **Formula** | **Range** | **Description** | **References** |
| --- | --- | --- | --- | --- | --- |
| Allelic frecuency | Allele Frequency (Codom Data) | $F_{x}=\frac{{2N}_{\mathrm{xx}}+N_{\mathrm{xy}}}{2N}$ | [0-1] | Calculated for a single locus. Determined for each allele. Nxx = # of XX homozygous individuals, and Nxy = # of XY heterozygous individuals, where Y can be any other allele. N = the number of samples. Can also be determined simply by direct count of the proportion of different alleles. | (Hartl and Clark, 1997) |
| Genotypic frequency | Genotypic frequency  (Haploid Data) | $F_{x}=\frac{N_{X}}{N}$ | [0-1] | Calculated for single loci, and determined for each allele, where Nx = number of the x alleles and N = the number of samples. Can also be determined by direct count of the proportion of different alleles. | (Hedrick, 2011) |
| Hardy-Weinberg disequilibrium | Chi-Squared  Test for HWE | $X^{2}=\sum_{i=1}^{k} \frac{\left( O-E \right)^{2}}{E}$ | [0,∞] | Where Oi is the observed number of individuals of the ith genotype, and Ei the expected number with DF = [Na(Na-1)]/2, where Na is the number of alleles at the locus. | (Hedrick, 2011) |
| Number of alleles | Allele  Frequency  (Binary Data) | Assuming random mating:  Presence = AA or Aa  Absence = aa.  Allele A has Freq p = 1 - q  Allele a has Freq q = 1 - p  Freq. of genotype aa  = q2 = Freq. of band absence  = 1- Freq. of band presence,  so q = √(Freq. of absence) | - | With dominant binary markers (e.g. AFLPs), it is not possible to directly calculate allele frequencies. If, we can assume either complete outcrossing (most animals and some plants) or obligate selfing (some agricultural plants), we can still estimate the allele frequencies. The basis of the GenAlEx estimate is shown to the left. Following Lynch and Milligan [80], it assumes complete outcrossing, but does not impose then recommended pruning of low frequency bands. Note: Zhivotovsky has developed an alternative Bayesian allele frequency estimation procedure that is available in other programs such as FAMD. GenAlExn6.3 onwards offers data export to this program. | (Lynch and Milligan, 1994) |
| Number of effective alleles | Effective  number of  alleles  (Haploid Data)  (Codom Data) | $N_{e}=\frac{1}{1-H_{e}}$  $cN_{e}= \frac{1}{1-H_{S}}$ | [1,n] | Here Ne represents an estimate of the number of equally frequent alleles in an ideal population. Enables meaningful comparisons of allelic diversity across loci with diverse allele frequency distributions. GenAlEx provides two slightly different estimates. The first (Ne) via Frequency is calculated by locus from HE for each population. The second (cNe) via G-statistics is calculated by locus over populations based on HS. | (Brown and Weir, 1983) |
| Shannon diversity index | Shannon diversity index | $I=-\sum p_{i}\ln p_{i}$ | - | Calculated on a single-locus basis, where ln = the natural logarithm and pi is the frequency of the ith allele. Equivalent to the Shannon-Weaver Index of ecology. Unlike He, not bounded by 1 and may therefore be a better measure of allelic and genetic diversity, though largely overlooked in genetic studies. GenAlEx 6.3 onwards also offers calculation of this and other Shannon indices for haploid and codominant data types via the Shannon options (see below). | (Brown and Weir, 1983) |
| Observed heterozygosity | Observed  Heterozygosity  (Codom Data) | $H_{0}=\frac{No of hets}{N}$ | - | Observed heterozygosity for a single locus within a population, where the number of heterozygotes is determined by direct count, N = sample size. | (Hedrick, 2011) |
| Expected heterozygosity | Expected  heterozygosity  (Codom Data) | $H_{e}=1-\sum{p_{i}}^{2}$ | [0,1] | HE is the Expected Heterozygosity or Genetic Diversity within a population. Calculated per locus as 1 minus the sum of the squared allele frequencies, pi 2. See also Mean He and Hs. | (Hartl and Clark, 1997) |
| Unbiased expected heterozygosity | Unbiased  Heterozygosity  (Codom Data) | $\mathrm{uH}_{E}=\frac{2n}{2n-1}\left( 1-\sum{p_{i}}^{2} \right)$ | [0,1] | An unbiased estimate of HE where pi is the frequency of the ith allele and n is the sample size. Often the only estimate of HE reported in other packages and should be the one reported from GenAlEx for research. HE is retained for teaching purposes. | (Peakall and Smouse, 2012) |
| Fixation index | Fixation Index  (Codom Data) | $F=\frac{H_{E}{-H}_{O}}{H_{E}}$ | [-1,1] | Calculated on a per locus basis. GenAlEx also provides the arithmetic mean across loci. Values close to zero are expected under random mating, while substantial positive values indicate inbreeding or undetected null alleles. Negative values indicate excess of heterozygosity, due to negative assortative mating, or heterotic selection | (Hartl and Clark, 1997) |
| Nei unbiased genetic distance | Nei’s Unbiased  Genetic Distance | $uD=-ln\left( \mathrm{uI} \right)$ | - | Nei’s unbiased genetic distance uD, where uI is the Unbiased Genetic Identity (see below for details) | (Hedrick, 2011) |
| Paired Fst | Linearized FST | $\mathrm{LinF}_{\mathrm{ST}}= \frac{F_{\mathrm{ST}}}{(1-F_{\mathrm{ST}})}$ | - | A transformation for pairwise population Fst values recommended by Slatkin [84]. GenAlEx also offers the analogous transformation for PhiPT. | (Slatkin, 1995) |

**Table S2. Genotypic and allelic frequencies of the CSN2 gene (A1 and A2 variants) in Holstein-Friesian, Jersey, Brown Swiss, Gyr and Cross-bred cattle breeds in different countries**

| **Country** | **n** | **A1A1** | **A1A2** | **A2A2** | **A1** | **A2** | **Year** | **Reference** |
| --- | --- | --- | --- | --- | --- | --- | --- | --- |
| **Holstein-Friesian** | | | | | | | | |
| Ecuador | 30 | 0.00 | 0.97 | 0.03 | 0.48 | 0.52 | 2024 | (Ruiz Alvarez and Salmerón Jiménez, 2024) |
| USA | 1,982 | 0.11 | 0.43 | 0.46 | 0.32 | 0.68 | 2023 | (Arens et al., 2023) |
| India | 61 | 0.42 | 0.52 | 0.04 | 0.69 | 0.31 | 2023 | (Khan et al., 2023) |
| Chile | 24 | - | - | - | 0.25 | 0.75 | 2023 | (Carvajal et al., 2023) |
| Peru | 102 | 0.20 | 0.46 | 0.34 | 0.43 | 0.57 | 2022 | (Zúñiga, 2022) |
| Spain | 1,868 | 0.21 | 0.48 | 031 | 0.45 | 0.55 | 2019 | (Alfonso et al., 2019) |
| Russia | 1,081 | 0.26 | 0.53 | 0.21 | 0.52 | 0.48 | 2021 | (Kovalyuk et al., 2021) |
| Turkey | 100 | 0.24 | 0.47 | 0.29 | 0.475 | 0.525 | 2022 | (Şahin & Boztepe, 2023) |
| Pakistan | 60 | 0.33 | 0.50 | 0.17 | 0.58 | 0.42 | 2022 | (Khan et al., 2023) |
| Slovakia | 1,478 | 0.052 | 0.370 | 0.576 | 0.238 | 0.761 | 2023 | (Miluchová et al., 2023) |
| Greece | 780 | 0.033 | 0.445 | 0.522 | 0.256 | 0.744 | 2021 | (Antonopoulos et al., 2021) |
| Croatia | 60 | 0.134 | 0.433 | 0.433 | 0.350 | 0.650 | 2021 | (Ivanković et al., 2021) |
| Italy (Midlands) | 1,629 | 0.132 | 0.458 | 0.410 | 0.361 | 0.639 | 2020 | (Sebastiani et al., 2020) |
| Italy (North) | 1,230 | 0.185 | 0.487 | 0.328 | 0.428 | 0.572 | 2017 | (Massella et al., 2017) |
| China | 133 | 0.271 | 0.444 | 0.285 | 0.492 | 0.508 | 2016 | (Dai et al., 2016) |
| Sweden | 415 | 0.089 | 0.446 | 0.465 | 0.312 | 0.688 | 2014 | (Gustavsson et al., 2014) |
| Ukraine | 747 | 0.183 | 0.447 | 0.37 | 0.406 | 0.594 | 2023 | (Ladyka et al., 2023) |
| **Jersey** | | | | | | | | |
| Ecuador | 27 | 0.04 | 0.96 | - | 0.52 | 0.48 | 2024 | (Ruiz Alvarez and Salmerón Jiménez, 2024) |
| India | 86 | 0.39 | 0.58 | 0.02 | 0.68 | 0.31 | 2023 | (Khan et al., 2023) |
| Chile | 24 | - | - | - | 0.27 | 0.73 | 2023 | (Carvajal et al., 2023) |
| Mexico | 453 | 0.04 | 0.29 | 0.43 | 0.19 | 0.71 | 2015 | (Zepeda-Batista et al., 2015) |
| Russia | 917 | 0.24 | 0.51 | 0.25 | 0.49 | 0.51 | 2021 | (Kovalyuk et al., 2021) |
| Turkey | 100 | 0.030 | 0.370 | 0.600 | 0.215 | 0.785 | 2022 | (Şahin and Boztepe, 2023) |
| Pakistan | 30 | 0.68 | 0.18 | 0.14 | 0.77 | 0.23 | 2022 | (Ayaz et al., 2023) |
| Japan | 590 | 0.032 | 0.304 | 0.664 | 0.184 | 0.816 | 2022 | (Nuomin et al., 2022) |
| Sweden | 406 | 0.108 | 0.382 | 0.510 | 0.299 | 0.701 | 2014 | (Gustavsson et al., 2014) |
| **Brown Swiss** | | | | | | | | |
| Ecuador | 29 | 0.03 | 0.97 | - | 0.52 | 0.48 | 2024 | (Ruiz Alvarez and Salmerón Jiménez, 2024) |
| Turkey | 100 | 0.15 | 0.44 | 0.41 | 0.37 | 0.63 | 2022 | (Şahin and Boztepe, 2023) |
| Ukraine | 145 | 0.086 | 0.486 | 0.428 | 0.329 | 0.671 | 2023 | (Ladyka et al., 2023) |
| Ukraine | 243 | 0.017 | 0.385 | 0.598 | 0.209 | 0.791 | 2023 | (Ladyka et al., 2023) |
| Croatia | 60 | 0.067 | 0.517 | 0.416 | 0.325 | 0.675 | 2021 | (Antonopoulos et al., 2021) |
| Slovenia | 190 | 0.026 | 0.352 | 0.621 | 0.203 | 0.797 | 2016 | (Potočnik et al., 2016) |
| **Gyr** | | | | | | | | |
| India | - | - | 0.07 | 0.93 | 0.04 | 0.96 | 2023 | (Khan et al., 2023) |
| Brazil | 593 | 0.02 | 0.19 | 0.79 | 0.11 | 0.89 | 2022 | (Pessoa et al., 2023) |
| Brazil | 68 | 0.00 | 0.44 | 0.956 | 0.022 | 0978 | 2017 | (Rangel et al., 2017) |
| **Crossbred (*Bos taurus*)** | | | | | | | | |
| Chile | 38 | - | - | - | 0.45 | 0.55 | 2023 | (Carvajal et al., 2023) |
